# Accuracy of the LaserSAFE technique for detecting positive surgical margins during robot‐assisted radical prostatectomy: blind assessment and inter‐rater agreement analysis

**DOI:** 10.1111/his.15336

**Published:** 2024-10-15

**Authors:** Ricardo Almeida‐Magana, Matthew Au, Tarek Al‐Hammouri, Manju Mathew, Kate Dinneen, Larissa S T Mendes, Eoin Dinneen, Willem Vreuls, Greg Shaw, Alex Freeman, Aiman Haider

**Affiliations:** ^1^ Department of Targeted Intervention University College London UK; ^2^ Department of Urology University College London Hospitals NHS Foundation Trust London UK; ^3^ Centre for Medical Imaging University College London UK; ^4^ Department of Histopathology University College London Hospitals NHS Foundation Trust London UK; ^5^ Department of Histopathology Canisius Wilhelmina Hospital Nijmegen the Netherlands; ^6^ LabPON Hengelo the Netherlands

**Keywords:** fluorescence confocal microscopy, frozen section analysis, margin assessment, prostate cancer

## Abstract

**Introduction and objectives:**

Fluorescence confocal microscopy (FCM) is a new imaging modality capable of generating digital microscopic resolution scans of fresh surgical specimens, and holds potential as an alternative to frozen section (FS) analysis for intra‐operative assessment of surgical margins. Previously, we described the LaserSAFE technique as an application of FCM for margin assessment in robot‐assisted radical prostatectomy (RARP) using the Histolog® scanner. This study describes the accuracy and inter‐rater agreement of FCM imaging compared to corresponding paraffin‐embedded analysis (PA) among four blinded pathologists for the presence of positive surgical margins (PSM).

**Materials and methods:**

RARP specimens from patients enrolled in the control arm of the NeuroSAFE PROOF study (NCT03317990) were analysed from April 2022 to February 2023. Prostate specimens were imaged using the Histolog® scanner before formalin fixation and PA. Four trained assessors, blinded to PA, reviewed and analysed FCM images of the posterolateral prostatic surface.

**Results:**

A total of 31 prostate specimens were included in the study. PA per lateral side of the prostate identified 11 instances of positive margins. Among the four histopathologists included in our study, FCM achieved a sensitivity of 73–91 and specificity of 94–100% for the presence of PSM. Fleiss’ Kappa for inter‐rater agreement on PSM was 0.78 (95% confidence interval = 0.64–0.92), indicating substantial agreement.

**Conclusion:**

This blinded analysis of FCM versus PA among histopathologists with different experience levels demonstrated high accuracy and substantial inter‐rater agreement for diagnosing PSM. This supports the role of the FCM as an alternative to FS.

## Graphical abstract

The LaserSAFE technique, based on fluorescence confocal microscopy to assess margins in radical prostatectomy, shows high accuracy in detecting positive surgical margins compared to paraffin‐embedded analysis.

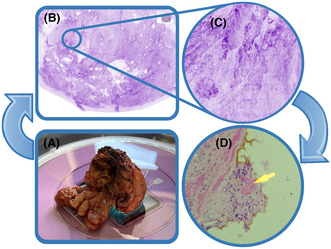

**Abbreviations**
BCR: Biochemical Recurrence
EPE: Extraprostatic Extension
FCM: Fluorescence Confocal Microscopy
FS: Frozen Section
IQR: Interquartile Range
ISUP: International Society of Urological Pathology
MRI: Magnetic Resonance Imaging
NS: Nerve Sparing
NVB: Neurovascular Bundle
PA: Paraffin‐embedded Analysis
PC: Prostate Cancer
PSA: Prostate‐Specific Antigen
PSM: Positive Surgical Margins
RARP: Robot‐Assisted Radical Prostatectomy

## Introduction

The goal of robot‐assisted radical prostatectomy (RARP) is the local oncological control of prostate cancer (PC). Risks associated with this procedure include postoperative urinary incontinence and erectile dysfunction.^1^, ^2^, ^3^ To decrease the incidence, severity and duration of these side effects, nerve‐sparing (NS) RARP was developed.^4^, ^5^ However, when extraprostatic extension (EPE) is present, NS poses an increased risk of positive surgical margins (PSM) at the posterolateral margins.^6^ PSM is a well‐recognised peri‐operative risk factor for biochemical recurrence (BCR)^7^ which, in turn, necessitates additional salvage treatments, with their associated distress, costs and negative impact on patients’ quality of life.^8^ The decision to perform NS is usually taken by the surgeon based on clinical information such as positive biopsy core locations, tumour extent on pre‐operative magnetic resonance imaging (MRI) and intra‐operative digital rectal examination, or a combination of these.^9^ However, the value of these tools in predicting EPE is limited, leading to the unnecessary removal of the neurovascular bundle in many patients.^10^, ^11^, ^12^

The NeuroSAFE technique, based on intra‐operative frozen section (FS) of the posterolateral margins, has emerged as a reliable guide for NS during RARP.^13^, ^14^, ^15^, ^16^ Despite its promise and adoption as standard in some centres,^14^ the NeuroSAFE technique is hindered from widespread implementation due to the cost and logistical requirements of performing FS.^17^ An alternative called *ex‐vivo* fluorescence confocal microscopy (FCM) is an emerging digital imaging technology capable of scanning fresh tissue to produce high‐resolution micrometre‐level images within minutes.^18^, ^19^ This technology has shown good early reliability and reproducibility at diagnosing prostate cancer in core biopsies and posterolateral shavings of the prostate similar to the NeuroSAFE technique.^20^, ^21^, ^22^

The Histolog® scanner (SamanTree Medical SA, Lausanne, Switzerland) is an *ex‐vivo* FCM‐based mobile microscope that can be placed within the operating room and has a wide objective capable of producing high‐resolution cellular‐level detail of fresh surgical specimens (48 × 36 in less than 1 min). We have previously described a technique to use the Histolog® scanner to image the posterolateral margins of RARP specimens which we have called LaserSAFE, as it is analogous to the NeuroSAFE FS technique.^23^ This method simplifies the procedure, reducing the number of steps by clearly protocolising specimen handling and thus allowing consistent good‐quality image production within minutes. In this paper, we describe the accuracy metrics of FCM images evaluated independently by four pathologists blinded to final pathology compared to the standard‐of‐care paraffin‐embedded analysis (PA).

## Methods

In this study, the specimens were evaluated using the Histolog® scanner through an *en‐face* technique; i.e. as seen from the outer aspect of the prostate and tangential to the area of interest. The primary objective was to describe the sensitivity of FCM image interpretation to diagnose PSM compared to the PA standard of care. Secondary objectives were to describe the specificity, positive and negative predictive values and interobserver agreement for the presence of PSM. Patients enrolled into the standard arm of the NeuroSAFE PROOF study (NCT03317990)^24^ who signed the optional consent form for prostate specimen biobanking analysis were included. RARP were performed from April 2022 to February 2023. All patients underwent standard‐of‐care RARP with NS decisions based on pre‐operative planning through MRI and biopsy information, and no clinical decisions were taken based on the findings of specimen analysis with FCM.

The RARP specimens of patients undergoing bilateral NS were imaged on the Histolog scanner® immediately after extraction from the abdominal cavity using our LaserSAFE technique. Briefly, the whole prostate is dipped for 10 s into an acridine orange‐based photoreactive solution (Histolog® Dip, SamanTree Medical SA) and rinsed using 0.9% saline solution, thoroughly dried using gauze and placed on the scanner to obtain three images: posterior, right posterolateral and left posterolateral. The scans were pseudo‐anonymised and saved in a secure hard drive for subsequent analysis. All scans were acquired by a urology research fellow (R.A.) trained in the use of the Histolog scanner. When irregularly shaped specimens precluded obtaining a high‐quality scan, a second image was acquired by using a light mouldable weight to compress and stabilise the specimen. Additionally, if the specimen volume was too large to fit within the Histolog scanner's objective, up to two images per side were acquired by repositioning the specimen.

Given the low rate of PSM in our current practice, we decided to enrich our population with *ex‐vivo*‐created PSMs. To this end, in patients where pre‐operative clinical and imaging parameters suggested a high likelihood of EPE or high‐volume intraprostatic malignancy, we performed an *ex‐vivo* dissection of the neurovascular bundle and Denonvilliers’ fascia to expose the prostatic capsule for imaging (peel technique, Supporting information, Video S1). This technique increased the likelihood of malignant glands being present at the surface of the specimen. The inner exposed area was subsequently imaged using the Histolog® scanner as described above, inked, and the specimen continuity was restored using cyanoacrylate‐based surgical glue to avoid affecting PA assessment of specimen and surgical margin status.

**Figure 1:**
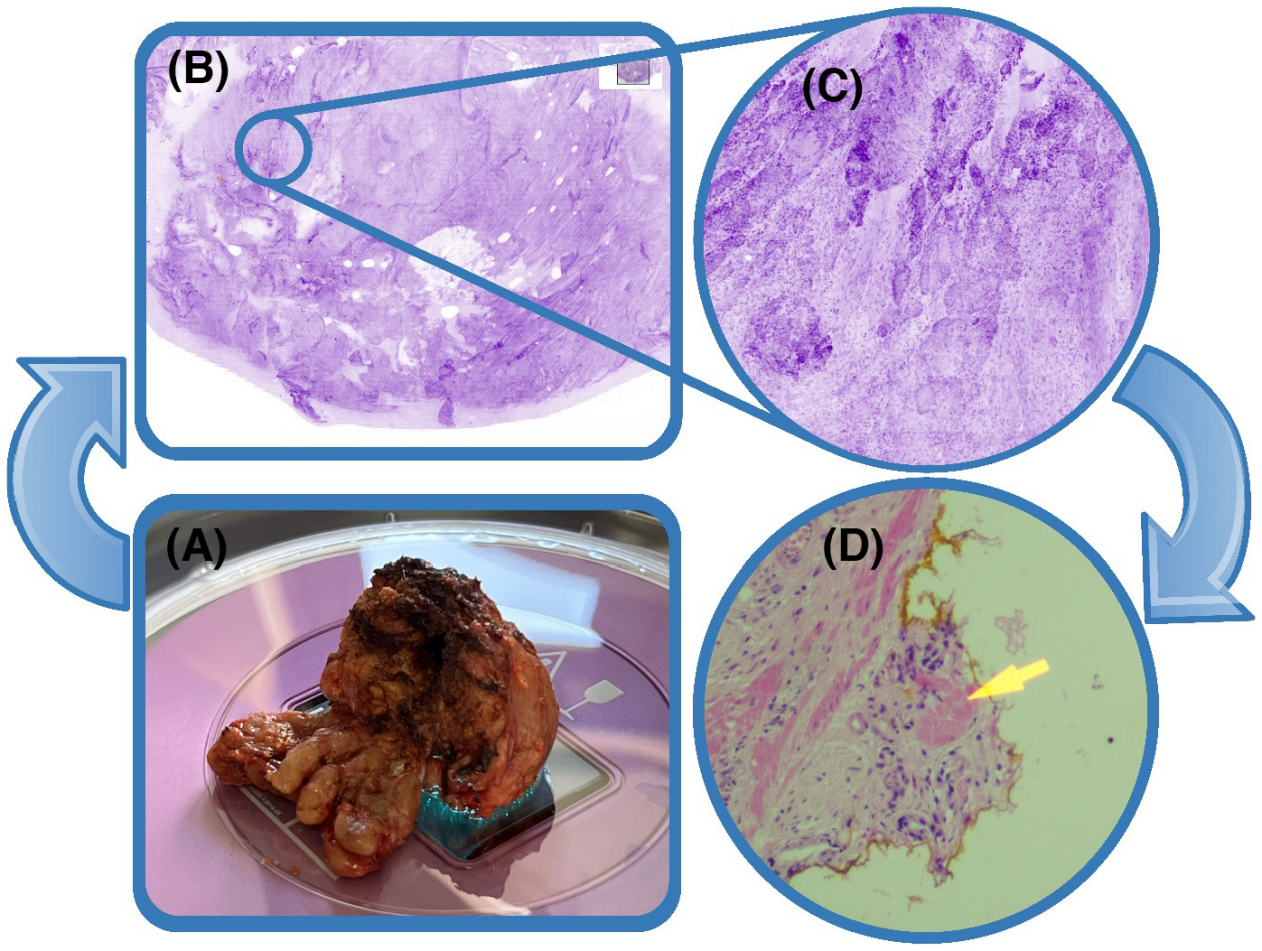
**A,** The prostate specimen is placed with the area of interest in contact with the microscope objective and stabilised using a purposely built specimen holder. **B,** The macroscopic high‐resolution image can be scanned for areas of interest. **C,** Magnification of glandular structures suggestive of a positive margin. **D,** Corresponding paraffin‐embedded haematoxylin and eosin image with malignant glands in contact with the ink. [Color figure can be viewed at wileyonlinelibrary.com]

The specimens were then formalin‐fixed and processed in the histopathology laboratory following routine practice and published guidelines, including inking the external specimen aspects for definite surgical margin assessment. All prostates were processed using the whole mount methodology.^25^, ^26^ The surgical margin was considered positive/involved when malignant glands were seen in contact with the inked surface of the specimen (Figure 1). When the *ex‐vivo* peel technique was performed on the prostate specimen for comparison with FCM, the surgical margin was considered positive if malignant glands were in contact with the inked internalised artificial surgical margin. As this study focuses upon the diagnostic accuracy of FCM, intra‐operative FCM margin status did not influence the surgical procedure. Furthermore, neither the artificial margin status nor the definitive surgical margin status affected adjuvant or salvage treatment decisions, as all patients were placed on PSA surveillance.

Four pathologists, including two consultant pathologists (A.F., W.V.) with expertise in uropathology and two pathology trainees (M.M., K.D.), assessed the FCM images. Before viewing the images, all pathologists received comprehensive training in FCM image interpretation provided through a dedicated training platform (https://hit.samantree.com/). One senior pathologist had previous experience with interpreting FCM imaging from a separate study (W.V.).

Following training, the pathologists independently reviewed the FCM images using a case report form (Supporting information, Figures S1 and S2) to identify and describe PSM. All evaluations were conducted retrospectively using an image bank and the Histolog Viewer software, which enables the review of Histolog scans on a personal desktop computer. Each patient had separate reports for the right and left sides, with the posterior image divided along the midline to correspond with the respective posterolateral sides. Evaluations were performed while blinded to both the final pathology results and the assessments of other participating pathologists. The decision to recommend secondary resection per side was left to the subjective opinion of the evaluator. Although we initially aimed to report Gleason grading in positive FCM cases as a secondary objective, this was hindered by the limitations of the Histolog technology, such as low image resolution at high magnification. Only two evaluators attempted to provide Gleason grading, but both expressed low confidence in their assessments. As a result, these findings have not been included in the manuscript. All results were collected in a dedicated database and analysed using R version 4.3.2, packages epiR and irrCAC.

## Results

**Figure 2:**
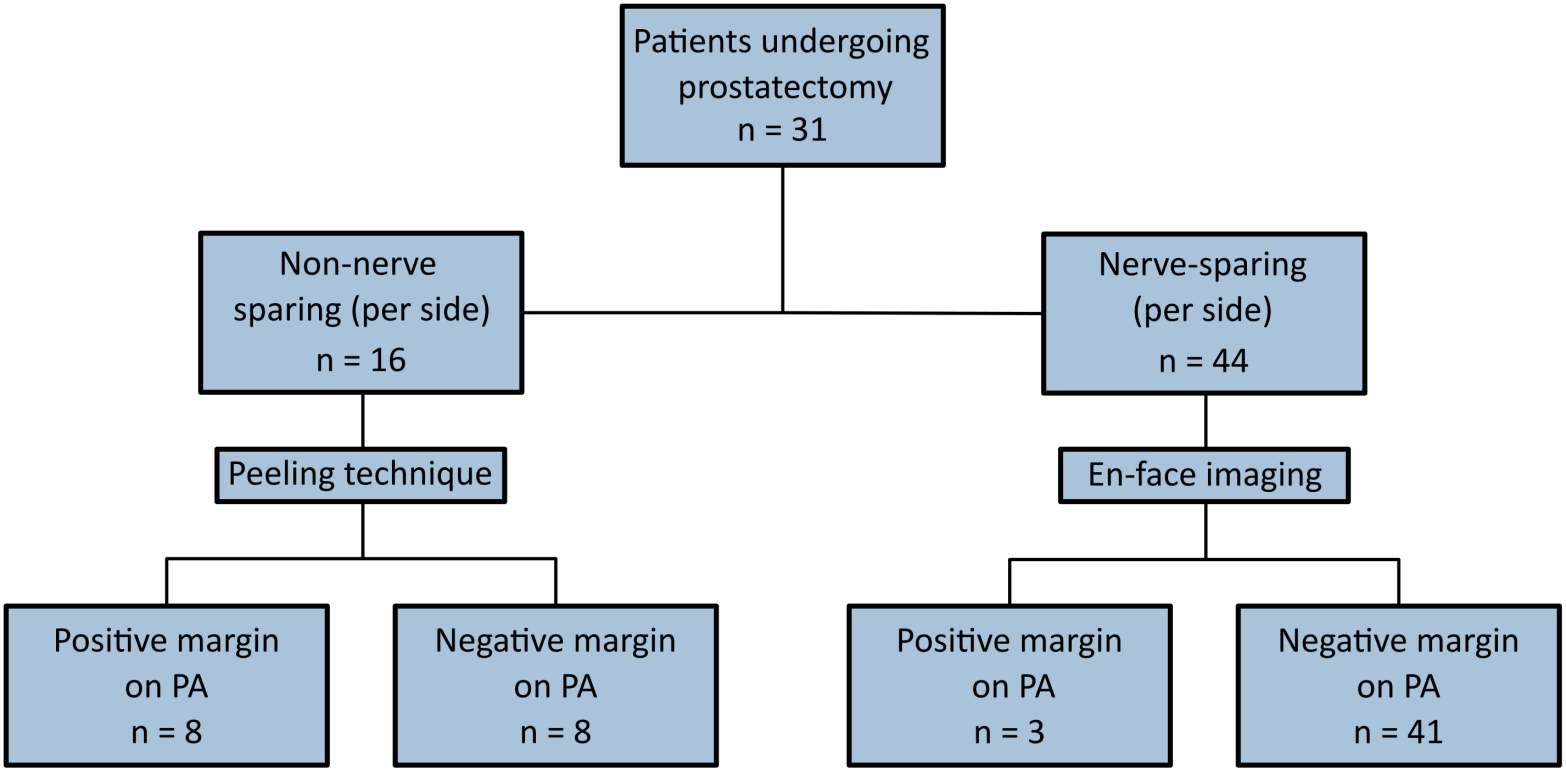
Distribution of cases according to the technique used to acquire fluorescence confocal microscopy images. [Color figure can be viewed at wileyonlinelibrary.com]

We performed the LaserSAFE image analysis on 31 patients. Table 1 shows the main clinical baseline and final pathological characteristics of the patients. Most cases were intermediate‐risk according to the EAU risk classification.^27^ In two cases, due to a protocol deviation, only one side of the prostate was scanned, resulting in 60 FCM analysable images. In 15 patients where a non‐NS surgery was performed on at least one side (one bilaterally), we performed the peel technique, which resulted in eight artificially created PSMs (Figure 2). Two patients had bilateral PSM on PA, resulting in a total of 11 prostate sides with PSM on PA. All pathologists assessed the quality of FCM images, including staining and artefacts, as adequate for diagnosis in all cases.

**Table 1:** Baseline characteristics per patient

Variable	*N* = 31
Age (years), median (IQR)	55 (49.50–59.5)
PSA at diagnosis (ng/dl), Median (IQR)	5 (4.05–9.6)
Prostate volume on MRI (cc), Median (IQR)	35 (24.50–45.0)
50–99 cc (%)	4 (11.4%)
>100 cc (%)	2 (5.7%)
Extraprostatic extension on MRI, *n* (%)	3 (9.7%)
Biopsy ISUP, *n* (%)	
2	23 (74.2%)
3	6 (19.4%)
4	1 (3.2%)
5	1 (3.2%)
Gland weight (g), median (IQR)	42 (38.00–52.0)
Extraprostatic extension on paraffin analysis, *n* (%)	9 (29.0%)
Final ISUP grade on paraffin analysis, *n* (%)	
2	26 (83.9%)
3	5 (16.1%)
Positive margin on paraffin analysis, *n* (%)	9 (29.0%)*
Tumour volume (ml), Median (IQR)	3 (2.32–4.0)

IQR, interquartile range; PSA, prostate specific antigen; MRI, magnetic resonance imaging; ISUP, International Society of Urological Pathology.

* Two patients had bilateral positive margins.

Table 2 illustrates the accuracy metrics for the presence of PSM by the four reviewers. Sensitivity ranged from 73 to 91%, while specificity ranged from 94 to 100% for the presence of PSM. The length of PSM measured on FCM ranged from 1 to 22 mm and findings were consistent among all reviewers (Supporting information, Table S1). The Fleiss‐Kappa agreement between all reviewers for the presence of PSM was 0.779 [95% confidence interval (CI) = 0.635–0.923] and for a clinical recommendation on resection of the neurovascular bundle 0.748 (95% CI = 0.577–0.92).

**Table 2:** Concordance between PA and FCM review per side

	Paraffin margin status			Accuracy metrics		
	Positive	Negative	Total	Sensitivity	Specificity	Kappa coefficient
Reviewer 1						
Positive	10	1	11	0.91 (0.59–1)	0.98 (0.59–1)	0.89 (0.73–1)
Negative	1	48	49			
Reviewer 2						
Positive	10	3	13	0.91 (0.59–1)	0.94 (0.83–0.99)	0.79 (0.53–0.99)
Negative	1	46	47			
Reviewer 3						
Positive	8	1	9	0.73 (0.39–0.94)	0.98 (0.89–1)	0.76 (0.53–0.98)
Negative	3	48	51			
Reviewer 4						
Positive	8	0	8	0.73 (0.39–0.94)	1.00 (0.93–1)	0.81 (0.60–1)
Negative	3	49	52			
Total	11	49	60			

*Note*: Green shading hightlights true positives on FCM.

Regarding false negative FCM cases, all reviewers missed the same PSM in a 148‐g prostate confirmed on PA as Gleason grade 3 + 3 for 3 mm. Additionally, two reviewers missed two separate PSMs on PA each measuring less than 3 mm. Three pathologists reported false positive findings on FCM, primarily attributable to small foci of ganglion cells mimicking glandular tissue appearance. In one instance, the presence of benign glands at the margin was confirmed by PA.

## Discussion

To the best of our knowledge, this paper is the first prospective evaluation of pathologists’ accuracy in interpreting *en‐face* FCM images of prostatectomy specimens while blinded to conventional cross‐sectional margin status. Promisingly, we observed a sensitivity ranging from 73 to 91% for diagnosing PSM among four observers. Other authors exploring FCM have reported similar findings.^28^ For instance, Baas *et al*.^22^ utilised the Histolog scanner on NeuroSAFE‐like prostate shavings and reported a sensitivity of 86% and specificity of 96%, aligning closely with our results. In conjunction, this strongly supports that FCM image interpretation is accurate and reliable in diagnosing PSM.

Rocco *et al*.^29^ have described the use of a different FCM‐based scanner (Vivascope 2500) with a Mohs‐like technique. In their study, when a PSM was detected a partial secondary resection of the suspect area was triggered. While we believe this method provides an interesting alternative to our technique, a drawback is that it requires up to 5 min per sample and the scan area is only 25 × 25 mm. In contrast, in our study, analysing the entire posterolateral surface in one step took 50 s per side.

Furthermore, previous descriptions of fluorescence confocal microscopy (FCM) technology require cutting the specimen to analyse the posterolateral margins, which presents three challenges: capsular retraction can falsely expose glandular tissue, potentially leading to false positives;^30^ the procedure requires a pathologist or an experienced technician to avoid damaging the specimen; and small samples may miss positive surgical margins (PSM) outside the sampled area. In contrast, the LaserSAFE technique allows for the analysis of the entire posterolateral prostatic surface without the need for cutting. Image acquisition can be performed without pathological or surgical expertise, consistently producing high‐quality images with minimal training.

Prior investigations into FCM within the context of RARP were constrained by a low prevalence of PSM in the cohorts.^29^, ^31^ In a systematic review describing the PSM rate during RARP, the mean prevalence of PSM was 15%.^32^ This low event rate hindered the generation of precise estimates for positive predictive value and sensitivity.^28^ The peel technique described here allowed us to observe an overall PSM rate of 29% without compromising oncological outcomes. This proved valuable in enriching our population, facilitating a more accurate assessment of the technique's efficacy in ruling PSM.

It is important to recognise that while beneficial for this study, the peel technique may produce images that are not fully representative of margins likely to be observed in real‐life practice, where PSMs may be less extensive. Nevertheless, in the two cases where a PSM was observed without the peeling technique, the length of malignant glands exposed was morphologically similar to those created by the peeling technique, albeit smaller in size (Supporting information, Figures S1 and S2).

Despite the associated disruption of the prostate, careful dissection and the application of additional ink to demarcate the artificially created margin were employed to prevent adverse effects on patient care. This precaution was ensured by having all RARP PA cases evaluated by a pathologist not involved in the blinded assessment. Furthermore, adjuvant treatments based on histological features are not currently standard of care at our institution,^33^ thus limiting the potential impact on patient treatment.

## Limitations

Limitations of this study include the small number of patients and the absence of a prespecified sample size, which affects the precision of our accuracy metrics. However, the use of multiple evaluators enhances our confidence in the reproducibility of the results. Another limitation is the lack of a formal evaluation of the learning curve for FCM image interpretation. Despite this, the comparable accuracy levels achieved by pathologists of varying experience suggest that the learning curve for identifying malignant glands is not steep. Developing annotated image banks such as the Atlas published by Bertoni *et al*.^34^ will aid in disseminating the expertise required to interpret these images. Furthermore, we advise caution when relying on FCM for prostates larger than 100 g, as this was the only instance where a PSM was missed by all evaluators, due probably to incomplete image capture during the FCM scan.

Interest in FCM for margin assessment is growing and we eagerly await the results of the fully powered IP‐8 study,^35^ which will evaluate the accuracy of whole gland imaging against PA. One of the promising advantages of FCM is its ability to allow remote image interpretation by pathologists. This eliminates the need to transport samples between facilities. This capability is particularly appealing in the current environment of limited time and personnel resources, which has impeded the widespread adoption of FS analysis by many centres.^36^ Additionally, the use of digital images opens the door for the development of artificial intelligence algorithms, which could further streamline the diagnostic process or assist in triage, enhancing efficiency even more. Our study aligns with the development of surgical interventions IDEAL stage 2a,^37^ and we consider the LaserSAFE technique to be fully developed and ready for evaluation in a prospective multicentre controlled trial. Therefore, we are conducting a feasibility study to compare the LaserSAFE technique with NeuroSAFE (Clinicaltrials.gov NCT06398470). These studies will provide definitive evidence on how this technology can be incorporated into clinical practice.

## Conclusion

In this blinded analysis of FCM‐based imaging of RARP specimens, we describe and further evaluate the LaserSAFE technique for fast intra‐operative analysis of the posterolateral NVB adjacent prostate margin. We report high sensitivity and specificity for detecting PSM among all reviewers and substantial interobserver agreement. These findings support the potential of the LaserSAFE technique as an alternative to NeuroSAFE and underscore the need for prospective studies to compare both methods head‐to‐head.

## Conflicts of interest

R.A.M. receives a salary from NIHR RFPB PB‐PG‐1216‐20013 and PCUK MA‐CT20‐11. G.S. receives consulting fees from Angle plc and has received travel expenses from Janssen and Johnson and Johnson (not related to the present manuscript). M.A., T.Al‐H., K.D, L.S.T.M., E.D., W.V., A.F. and A.H. report no conflicts of interest related to this work. SamanTree Medical SA (Lausanne, Switzerland) provided the Histolog® scanner, training and consumables but was not involved in the study design, execution, results or writing of the manuscript.

## Patient consent

All patients who participated in the study understood and signed the informed consent form for the NeuroSAFE PROOF Study and Optional Biobanking Consent for the UCLH site.

## Supporting information


**Video S1.** Video description of the peel technique and FCM imaging process.

**Figure S1.** Comparison between a PSM encountered during standard specimen processing 1.98 × 2.4 mm (A) and PSM created by the peel technique 2.6 × 5.1 mm (B).
**Figure S2.** Measured length of PSM identified on confocal microscopy across reviewers.

## Data Availability

The data that support the findings of this study are available on request from the corresponding author. The data are not publicly available due to privacy or ethical restrictions.

## References

[1] Corsini C , Bergengren O , Carlsson S *et al*. Patient‐reported side effects 1 year after radical prostatectomy or radiotherapy for prostate cancer: a register‐based Nationwide study. Eur. Urol. Oncol. 2024; 7; 605–613. 38233329 10.1016/j.euo.2023.12.007 PMC11102330

[2] Averbeck MA , Marcelissen T , Anding R , Rahnama'I MS , Sahai A , Tubaro A . How can we prevent postprostatectomy urinary incontinence by patient selection, and by preoperative, peroperative, and postoperative measures? International Consultation on Incontinence‐Research Society 2018. Neurourol. Urodyn. 2019; 38(Suppl 5); S119–S126. 31821626 10.1002/nau.23972

[3] Nyberg M , Hugosson J , Wiklund P *et al*. Functional and oncologic outcomes between open and robotic radical prostatectomy at 24‐month follow‐up in the Swedish LAPPRO trial. Eur. Urol. Oncol. 2018; 1; 353–360. 31158073 10.1016/j.euo.2018.04.012 PMC7061692

[4] Nguyen LN , Head L , Witiuk K *et al*. The risks and benefits of cavernous neurovascular bundle sparing during radical prostatectomy: a systematic review and meta‐analysis. J. Urol. 2017; 198; 760–769. 28286069 10.1016/j.juro.2017.02.3344

[5] Walsh PC , Lepor H , Eggleston JC . Radical prostatectomy with preservation of sexual function: anatomical and pathological considerations. Prostate 1983; 4; 473–485. 6889192 10.1002/pros.2990040506

[6] Zhang L , Zhao H , Wu B , Zha Z , Yuan J , Feng Y . Predictive factors for positive surgical margins in patients with prostate cancer after radical prostatectomy: a systematic review and meta‐analysis. Front. Oncol. 2021; 10; 539592. 33628724 10.3389/fonc.2020.539592 PMC7897672

[7] Pellegrino F , Falagario UG , Knipper S *et al*. Assessing the impact of positive surgical margins on mortality in patients who underwent robotic radical prostatectomy: 20 Years' report from the EAU robotic urology section scientific working group. Eur. Urol. Oncol. 2024; 7; 888–896. 38155061 10.1016/j.euo.2023.11.021

[8] Ost P , Lumen N , Goessaert A‐S *et al*. High‐dose salvage intensity‐modulated radiotherapy with or without androgen deprivation after radical prostatectomy for rising or persisting prostate‐specific antigen: 5‐year results. Eur. Urol. 2011; 60; 842–849. 21514039 10.1016/j.eururo.2011.04.021

[9] Asfuroğlu U , Asfuroğlu BB , Özer H *et al*. Which one is better for predicting extraprostatic extension on multiparametric MRI: ESUR score, Likert scale, tumor contact length, or EPE grade? Eur. J. Radiol. 2022; 149; 110228. 35255320 10.1016/j.ejrad.2022.110228

[10] Gandaglia G , Ploussard G , Valerio M *et al*. The key combined value of multiparametric magnetic resonance imaging, and magnetic resonance imaging‐targeted and concomitant systematic biopsies for the prediction of adverse pathological features in prostate cancer patients undergoing radical prostatectomy. Eur. Urol. 2020; 77; 733–741. 31547938 10.1016/j.eururo.2019.09.005

[11] Dinneen E , Allen C , Strange T *et al*. Negative mpMRI rules out extra‐prostatic extension in prostate cancer before robot‐assisted radical prostatectomy. Diagnostics (Basel) 2022; 12; 1057. 35626214 10.3390/diagnostics12051057 PMC9139507

[12] Rocco B , Sighinolfi MC , Sandri M *et al*. Is Extraprostatic extension of cancer predictable? A review of predictive tools and an external validation based on a large and a single center cohort of prostate cancer patients. Urology 2019; 129; 8–20. 30928608 10.1016/j.urology.2019.03.019

[13] Eichelberg C , Erbersdobler A , Haese A *et al*. Frozen section for the management of intraoperatively detected palpable tumor lesions during nerve‐sparing scheduled radical prostatectomy. Eur. Urol. 2006; 49; 1011–1016. 16546316 10.1016/j.eururo.2006.02.035

[14] Schlomm T , Tennstedt P , Huxhold C *et al*. Neurovascular structure‐adjacent frozen‐section examination (NeuroSAFE) increases nerve‐sparing frequency and reduces positive surgical margins in open and robot‐assisted laparoscopic radical prostatectomy: experience after 11,069 consecutive patients. Eur. Urol. 2012; 62; 333–340. 22591631 10.1016/j.eururo.2012.04.057

[15] van der Slot MA , Remmers S , van Leenders G *et al*. Urinary incontinence and sexual function after the introduction of NeuroSAFE in radical prostatectomy for prostate cancer. Eur. Urol. Focus 2023; 9; 824–831. 37032279 10.1016/j.euf.2023.03.021

[16] Dinneen EP , Van Der Slot M , Adasonla K *et al*. Intraoperative frozen section for margin evaluation during radical prostatectomy: a systematic review. Eur. Urol. Focus 2020; 6; 664–673. 31787570 10.1016/j.euf.2019.11.009

[17] Gretser S , Hoeh B , Kinzler MN *et al*. The NeuroSAFE frozen section technique during radical prostatectomy – implementation and optimization of technical aspects in a routine pathology workflow. Pathol. Res. Pract. 2023; 242; 154297. 36621159 10.1016/j.prp.2022.154297

[18] Gareau DS , Jeon H , Nehal KS , Rajadhyaksha M . Rapid screening of cancer margins in tissue with multimodal confocal microscopy. J. Surg. Res. 2012; 178; 533–538. 22721570 10.1016/j.jss.2012.05.059 PMC3458153

[19] Lauwerends LJ , van Driel P , Baatenburg de Jong RJ *et al*. Real‐time fluorescence imaging in intraoperative decision making for cancer surgery. Lancet Oncol. 2021; 22; e186–e195. 33765422 10.1016/S1470-2045(20)30600-8

[20] Puliatti S , Bertoni L , Pirola GM *et al*. Ex vivo fluorescence confocal microscopy: the first application for real‐time pathological examination of prostatic tissue. BJU Int. 2019; 124; 469–476. 30908852 10.1111/bju.14754

[21] Rocco B , Sighinolfi MC , Sandri M *et al*. Digital biopsy with fluorescence confocal microscope for effective real‐time diagnosis of prostate cancer: a prospective, comparative study. Eur. Urol. Oncol. 2021; 4; 784–791. 32952095 10.1016/j.euo.2020.08.009

[22] Baas DJH , Vreuls W , Sedelaar JPMM *et al*. Confocal laser microscopy for assessment of surgical margins during radical prostatectomy. BJU Int. 2023; 132; 40–46. 36440864 10.1111/bju.15938

[23] Almeida‐Magana R , Au M , Al‐Hammouri T *et al*. Improving fluorescence confocal microscopy for margin assessment during robot‐assisted radical prostatectomy: the LaserSAFE technique. BJU Int. 2024; 133; 677–679. 38009389 10.1111/bju.16239

[24] Dinneen E , Grierson J , Almeida‐Magana R *et al*. NeuroSAFE PROOF: study protocol for a single‐blinded, IDEAL stage 3, multi‐centre, randomised controlled trial of NeuroSAFE robotic‐assisted radical prostatectomy versus standard robotic‐assisted radical prostatectomy in men with localized prostate cancer. Trials 2022; 23; 584. 35869497 10.1186/s13063-022-06421-7 PMC9306247

[25] Kench JG , Judge M , Delahunt B *et al*. Dataset for the reporting of prostate carcinoma in radical prostatectomy specimens: updated recommendations from the International Collaboration on Cancer Reporting. Virchows Arch. 2019; 475; 263–277. 31098802 10.1007/s00428-019-02574-0

[26] Samaratunga H , Montironi R , True L *et al*. International Society of Urological Pathology (ISUP) consensus conference on handling and staging of radical prostatectomy specimens. Working group 1: specimen handling. Mod. Pathol. 2011; 24; 6–15. 20834234 10.1038/modpathol.2010.178

[27] Cornford P , van den Bergh RCN , Briers E *et al*. EAU‐EANM‐ESTRO‐ESUR‐ISUP‐SIOG guidelines on prostate Cancer‐2024 update. Part I: screening, diagnosis, and local treatment with curative intent. Eur. Urol. 2024; 86; 148–163. 38614820 10.1016/j.eururo.2024.03.027

[28] Au M , Almeida‐Magana R , Al‐Hammouri T , Haider A , Shaw G . A systematic review. J. Histochem. Cytochem. 2023; 71; 661–674. 37968920 10.1369/00221554231212948 PMC10691410

[29] Rocco B , Sarchi L , Assumma S *et al*. Digital frozen sections with fluorescence confocal microscopy during robot‐assisted radical prostatectomy: surgical technique. Eur. Urol. 2021; 80; 724–729. 33965288 10.1016/j.eururo.2021.03.021

[30] Philippou Y , Harriss E , Davies L *et al*. Prostatic capsular incision during radical prostatectomy has important oncological implications: a systematic review and meta‐analysis. BJU Int. 2019; 124; 554–566. 30113754 10.1111/bju.14522

[31] Eissa A , Zoeir A , Sighinolfi MC *et al*. ‘Real‐time’ assessment of surgical margins during radical prostatectomy: state‐of‐the‐art. Clin. Genitourin. Cancer 2020; 18; 95–104. 31784282 10.1016/j.clgc.2019.07.012

[32] Novara G , Ficarra V , Mocellin S *et al*. Systematic review and meta‐analysis of studies reporting oncologic outcome after robot‐assisted radical prostatectomy. Eur. Urol. 2012; 62; 382–404. 22749851 10.1016/j.eururo.2012.05.047

[33] Vale CL , Fisher D , Kneebone A *et al*. Adjuvant or early salvage radiotherapy for the treatment of localised and locally advanced prostate cancer: a prospectively planned systematic review and meta‐analysis of aggregate data. Lancet 2020; 396; 1422–1431. 33002431 10.1016/S0140-6736(20)31952-8 PMC7611137

[34] Bertoni L , Puliatti S , Reggiani Bonetti L *et al*. Ex vivo fluorescence confocal microscopy: prostatic and periprostatic tissues atlas and evaluation of the learning curve. Virchows Arch. 2020; 476; 511–520. 31907606 10.1007/s00428-019-02738-y

[35] Mayor N. Imperial prostate 8 – fluorescence confocal microscopy for rapid evaluation of surgical cancer excision. London: ISRCTN, 2023. [Accessed Jun 14, 2024]. Available at: 10.1186/ISRCTN21536411

[36] Oxley J , Bray A , Rowe E . Could a Mohs technique make NeuroSAFE a viable option? BJU Int. 2018; 122; 358–359. 29727911 10.1111/bju.14377

[37] Mcculloch P , Altman DG , Campbell WB *et al*. No surgical innovation without evaluation: the IDEAL recommendations. Lancet 2009; 374; 1105–1112. 19782876 10.1016/S0140-6736(09)61116-8

