# Supplementary material for: Accuracy of the LaserSAFE technique for detecting positive surgical margins during robot‐assisted radical prostatectomy: blind assessment and inter‐rater agreement analysis
Source: Histopathology. 2024 Oct 15;86(3):433–40. doi: 10.1111/his.15336 (PMC11707496; doi:10.1111/his.15336)
Supplement: Supplementary file 2 — Figure S1. Comparison between a PSM encountered during standard specimen processing 1.98 × 2.4 mm (A) and PSM created by the peel technique 2.6 × 5.1 mm (B). Figure S2. Measured length of PSM identified on confocal microscopy across reviewers. [file HIS-86-433-s001.docx]

Figure S1

Legend: Comparison between a PSM encountered during standard specimen processing 1.98x2.4 mm (A) and PSM created by the peel technique 2.6x5.1 mm(B)


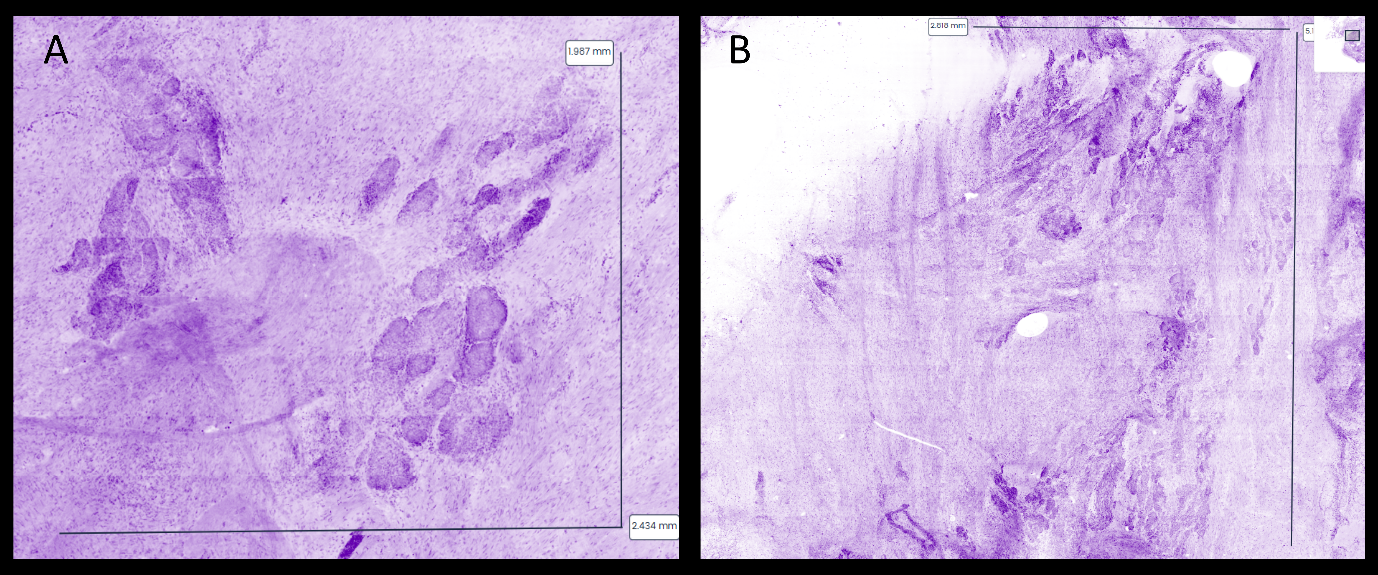


Figure S2

Legend: Measured length of PSM identified on confocal microscopy across reviewers

| Positive Margin identifier | Reviewer 1 | Reviewer 2 | Reviewer 3 | Reviewer 4 |  |
| --- | --- | --- | --- | --- | --- |
|  | Measured length (mm) | | | | Technique |
| 1 | 7.2 | 7.6 | 7 | 8 | Peel |
| 2 | 7.3 | 4.7 | 3.5 | 11 | Peel |
| 3 | 2 | 2 | 2 | 4 | En-face |
| 4 | 1.9 | 1.9 | 2 | 7 | En-face |
| 5 | 1.7 | 3.2 | - | - | Peel |
| 6 | 1 | 2.8 | 1 | 1.5 | Peel |
| 7 | 10.1 | 12 | 10 | 15 | Peel |
| 8 | 1.44 | 1.3 | - | - | Peel |
| 9 | 6.4 | 1 | 6 | 7 | En-face |
| 10 | 22.2 | 23 | 18 | 22 | Peel |

***LaserSAFE - to - Paraffin Concordance***

***CONFOCAL INFO CAPTURE SHEET***

Site:

Date: Patient ID:

Specimen out: LaserSAFE begins: LaserSAFE ends:

Description of the technique:

| Laterality | Left | Right |
| --- | --- | --- |
| Completeness Scan Tissue  (Air bubbles and surface) | Complete >90% ( )  Partial 50-89% ( )  Incomplete ( ) | Complete >90% ( )  Partial 50-89% ( )  Incomplete ( ) |
| Staining Quality  (Contrast between structures) | Good ( )  Poor ( ) | Good ( )  Poor ( ) |
| Glands visible | Yes ( ) No ( ) | Yes ( ) No ( ) |
| Nuclear Clarity  (In glandular tissue) | Good ( )  Poor ( ) | Good ( )  Poor ( ) |
| Margin status | Positive ( )  Negative ( )  Not assessable ( ) | Positive ( )  Negative ( )  Not assessable ( ) |
| Location of PSM | Apical ( )  Mid gland ( )  Base ( )  Multifocal ( ) | Apical ( )  Mid gland ( )  Base ( )  Multifocal ( ) |
| Cancer Grade | Gleason score ( )  Not assessable ( ) | Gleason score ( )  Not assessable ( ) |
| Length of PSM | _______ mm | _______ mm |
| Clinical recommendation | No resection ( )  Resect ipsilateral bundle ( ) | No resection ( )  Resect ipsilateral bundle ( ) |
| Comments |  |  |

***LaserSAFE - to - Paraffin Concordance***

***Paraffin info capture sheet***

Site:

Date: Patient ID:

| Laterality | Left ( )  Right ( ) |
| --- | --- |
| Specimen processing | Adequate ( )  Artifacts present ( )  Not assessable ( ) |
| Staining Quality | Good ( )  Partial ( )  Bad ( ) |
| Margin status (posterolateral only) | Positive ( )  Negative ( )  Narrowly clear ( ) |
| Location of PSM | Apical ( )  Mid gland ( )  Base ( )  Multifocal ( ) |
| Positive margin outside sampled area? | Yes ( ) No ( ) |
| Gleason score at the margin | Gleason score ( )  Not assessable ( ) |
| Length of PSM (Main lesion and sum) | _______ mm  _______ mm |
| Overall Gleason Score |  |
| Volume of tumour | _______ ml |
| EPE | Yes ( ) No ( ) |
|  |  |
|  |  |
|  |  |
